# Supplementary material for: Mutational Profile Enables the Identification of a High-Risk Subgroup in Myelodysplastic Syndromes with Isolated Trisomy 8
Source: Cancers (Basel). 2023 Jul 27;15(15):3822. doi: 10.3390/cancers15153822 (PMC10417840; doi:10.3390/cancers15153822)
Supplement: Supplementary file 1 [file cancers-15-03822-s001.zip › Supplementary figures.pptx]

## Slide 1
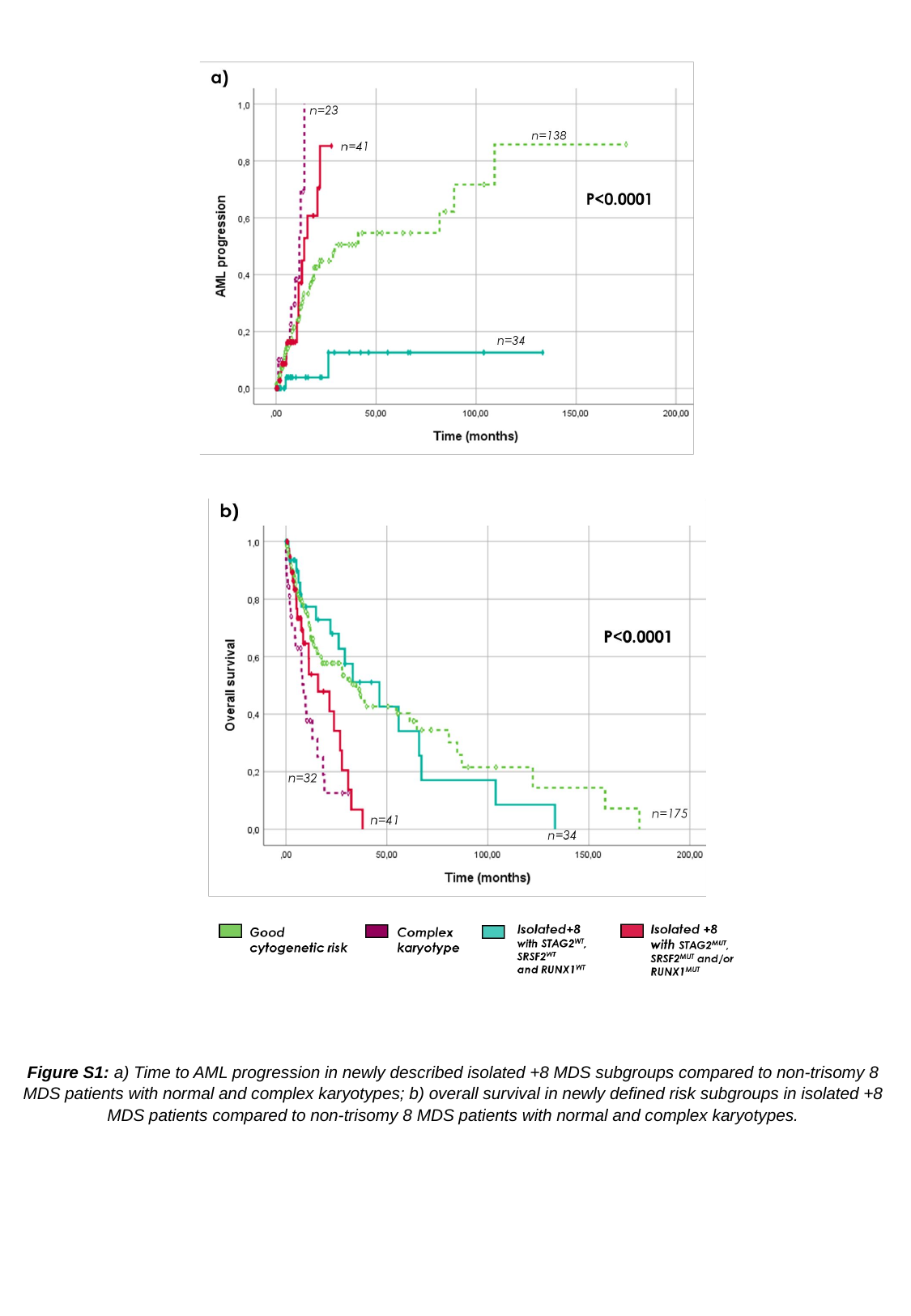

Figure S1: a) Time to AML progression in newly described isolated +8 MDS subgroups compared to non-trisomy 8 MDS patients with normal and complex karyotypes; b) overall survival in newly defined risk subgroups in isolated +8 MDS patients compared to non-trisomy 8 MDS patients with normal and complex karyotypes.

## Slide 2
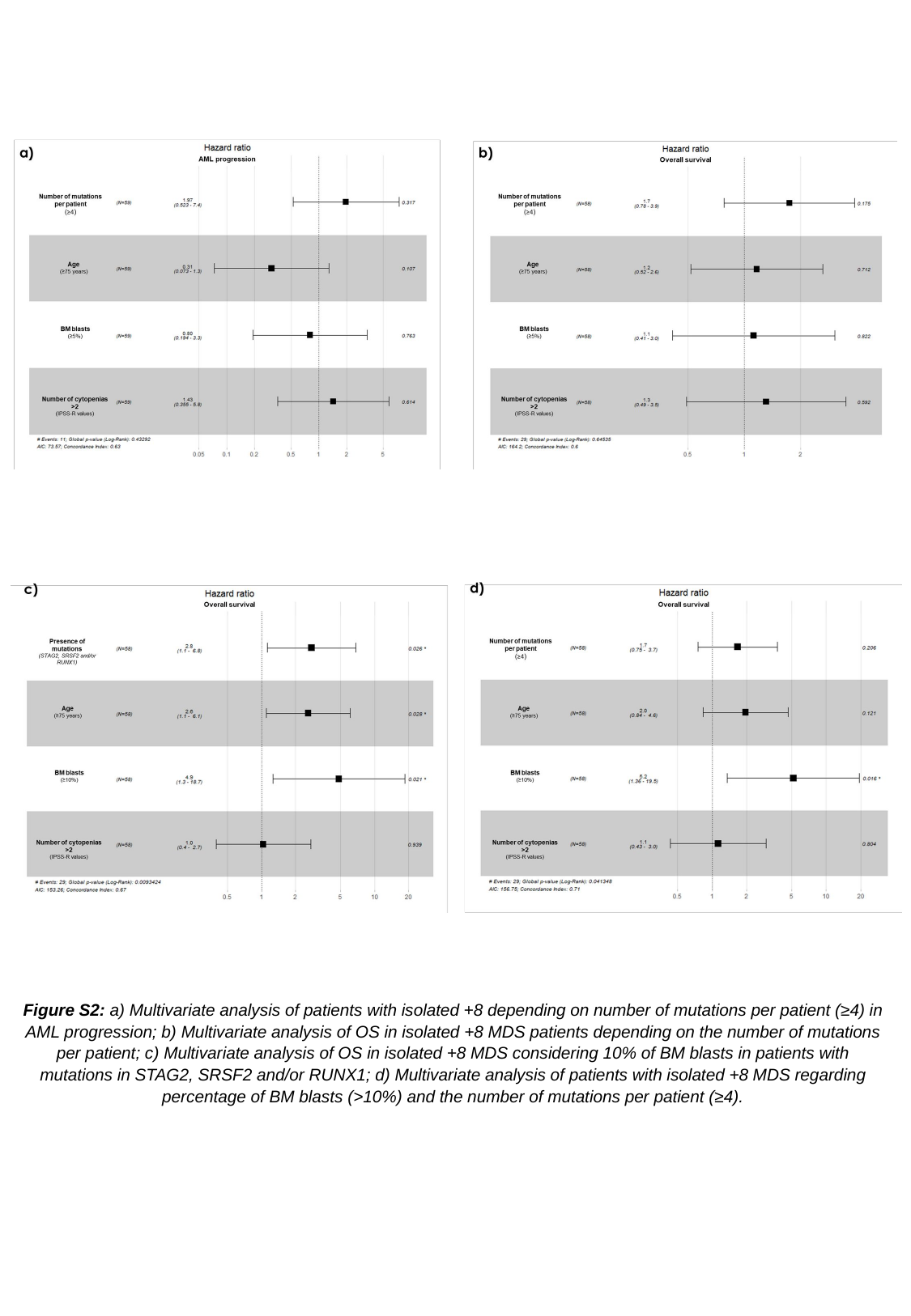

Figure S2: a) Multivariate analysis of patients with isolated +8 depending on number of mutations per patient (≥4) in AML progression; b) Multivariate analysis of OS in isolated +8 MDS patients depending on the number of mutations per patient; c) Multivariate analysis of OS in isolated +8 MDS considering 10% of BM blasts in patients with mutations in STAG2, SRSF2 and/or RUNX1; d) Multivariate analysis of patients with isolated +8 MDS regarding percentage of BM blasts (>10%) and the number of mutations per patient (≥4).

## Slide 3
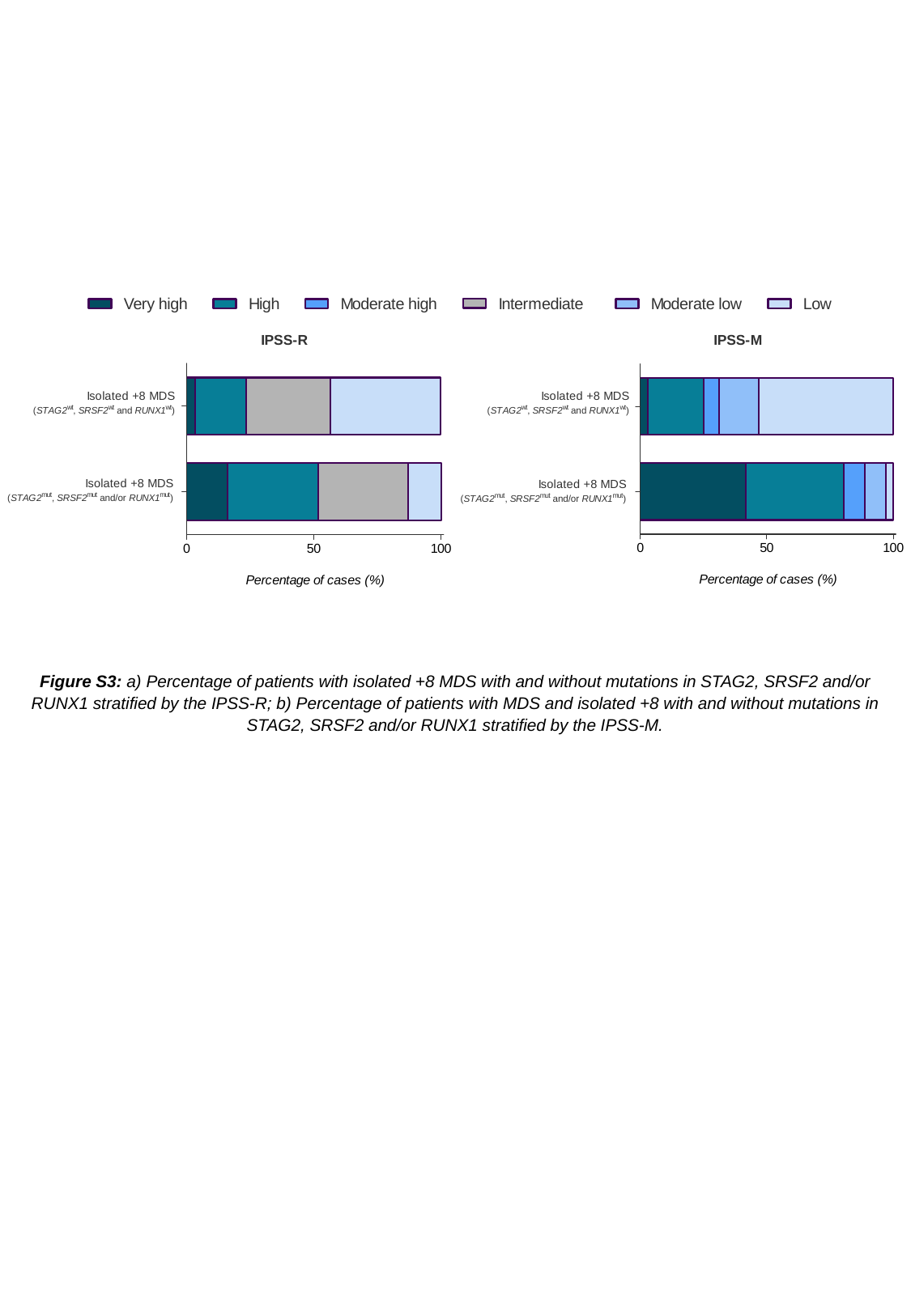

Figure S3: a) Percentage of patients with isolated +8 MDS with and without mutations in STAG2, SRSF2 and/or RUNX1 stratified by the IPSS-R; b) Percentage of patients with MDS and isolated +8 with and without mutations in STAG2, SRSF2 and/or RUNX1 stratified by the IPSS-M.

## Slide 4
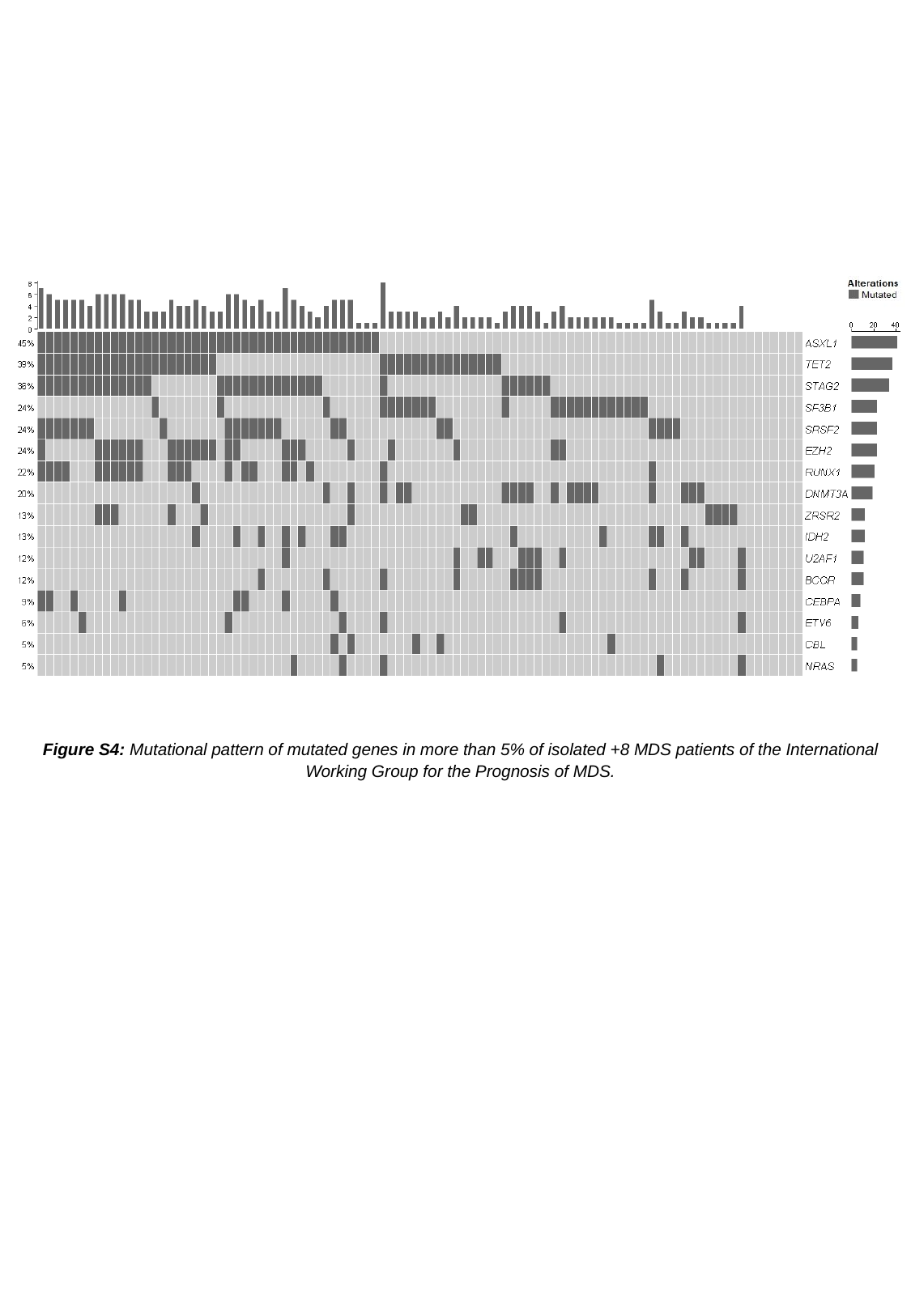

Figure S4: Mutational pattern of mutated genes in more than 5% of isolated +8 MDS patients of the International Working Group for the Prognosis of MDS.

## Slide 5
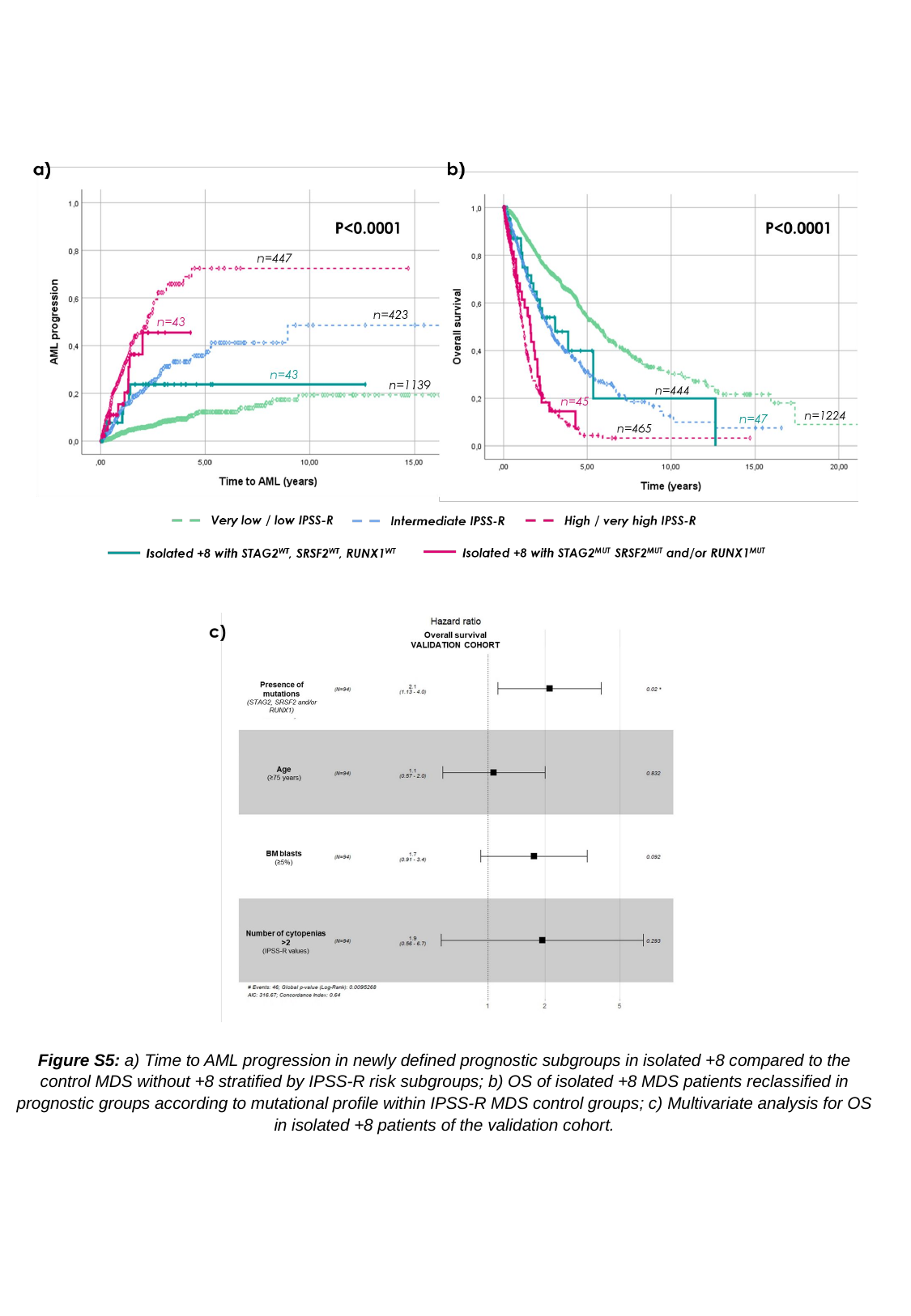

Figure S5: a) Time to AML progression in newly defined prognostic subgroups in isolated +8 compared to the control MDS without +8 stratified by IPSS-R risk subgroups; b) OS of isolated +8 MDS patients reclassified in prognostic groups according to mutational profile within IPSS-R MDS control groups; c) Multivariate analysis for OS in isolated +8 patients of the validation cohort.

## Slide 6
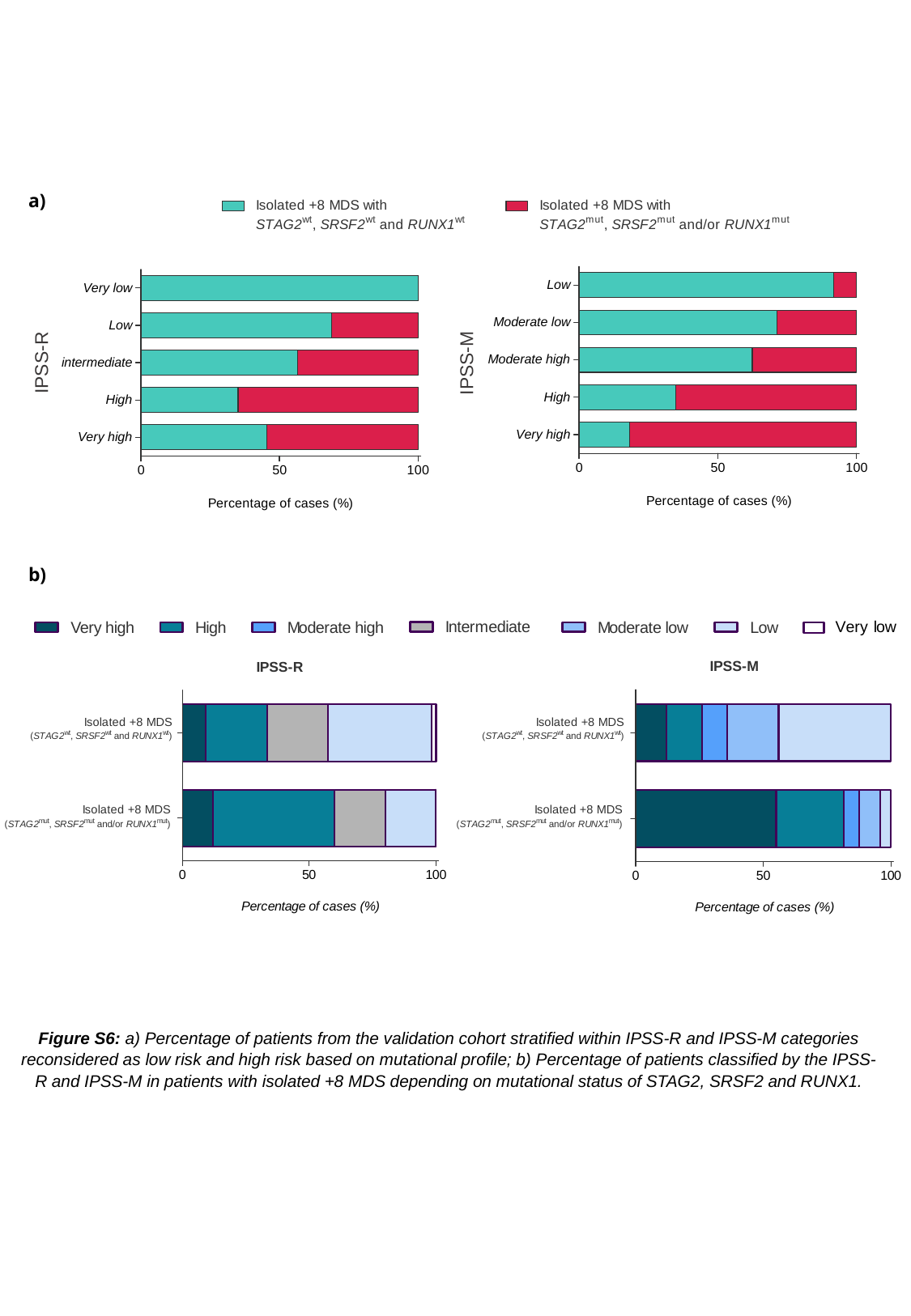

a)
IPSS-R
IPSS-M
b)
Figure S6: a) Percentage of patients from the validation cohort stratified within IPSS-R and IPSS-M categories reconsidered as low risk and high risk based on mutational profile; b) Percentage of patients classified by the IPSS-R and IPSS-M in patients with isolated +8 MDS depending on mutational status of STAG2, SRSF2 and RUNX1.
